# The protective effect of natural medicines against excessive inflammation and oxidative stress in acute lung injury by regulating the Nrf2 signaling pathway

**DOI:** 10.3389/fphar.2022.1039022

**Published:** 2022-11-16

**Authors:** Rumei Luan, Dongyan Ding, Junling Yang

**Affiliations:** Department of Respiratory Medicine, The Second Hospital of Jilin University, Changchun, China

**Keywords:** ali, Nrf2, oxidative stress, inflammation, natural medicine

## Abstract

Acute lung injury (ALI) is a common critical disease of the respiratory system that progresses into acute respiratory distress syndrome (ARDS), with high mortality, mainly related to pulmonary oxidative stress imbalance and severe inflammation. However, there are no clear and effective treatment strategies at present. Nuclear factor erythroid 2-related factor 2(Nrf2) is a transcription factor that interacts with multiple signaling pathways and regulates the activity of multiple oxidases (NOX, NOS, XO, CYP) related to inflammation and apoptosis, and exhibits antioxidant and anti-inflammatory roles in ALI. Recently, several studies have reported that the active ingredients of natural medicines show protective effects on ALI *via* the Nrf2 signaling pathway. In addition, they are cheap, naturally available, and possess minimal toxicity, thereby having good clinical research and application value. Herein, we summarized various studies on the protective effects of natural pharmaceutical components such as polyphenols, flavonoids, terpenoids, alkaloids, and polysaccharides on ALI through the Nrf2 signaling pathway and demonstrated existing gaps as well as future perspectives.

## 1 Introduction

Acute lung injury (ALI)/acute respiratory distress syndrome (ARDS) is caused by various intrapulmonary and extrapulmonary pathogenic factors, resulting in diffuse interstitial and alveolar edema, clinically presented as progressive hypoxemia and respiratory distress (Meyer et al., 2021). The pathogenesis of this disease has not been fully elucidated and is mainly influenced by an acute inflammatory response, excessive oxidative stress, apoptosis, and autophagy (Deng et al., 2020; Meyer et al., 2021). The current morbidity and mortality of ALI/ARDS are still high. Moreover, an international multicenter study on ARDS in 459 intensive care unit (ICU) patients from 50 countries demonstrated that the prevalence of ARDS was 10.4% in 29,144 patients admitted to the ICU, and the in-hospital mortality rate of patients with all forms of ARDS was 34.9% (Bellani et al., 2016). In addition, an international observational study involving 145 pediatric intensive care units (PICUs) demonstrated that the morbidity and mortality rates of ARDS were 3.2% and 17.1% in 23,280 children (Khemani et al., 2019). Currently, adjunctive therapy for patients with ALI/ARDS includes simvastatin (Grommes et al., 2012a), pioglitazone (Grommes et al., 2012b), and aspirin (Tilgner et al., 2016). These therapies can effectively reduce the inflammatory response in patients with ALI while also causing gastrointestinal or liver damage and other side effects (Abd El Aal et al., 2017; Chen et al., 2017). Therefore, the development of drugs with minimal toxicity will be of greater clinical utility in the prevention and treatment of ALI/ARDS.

**FIGURE 1:**
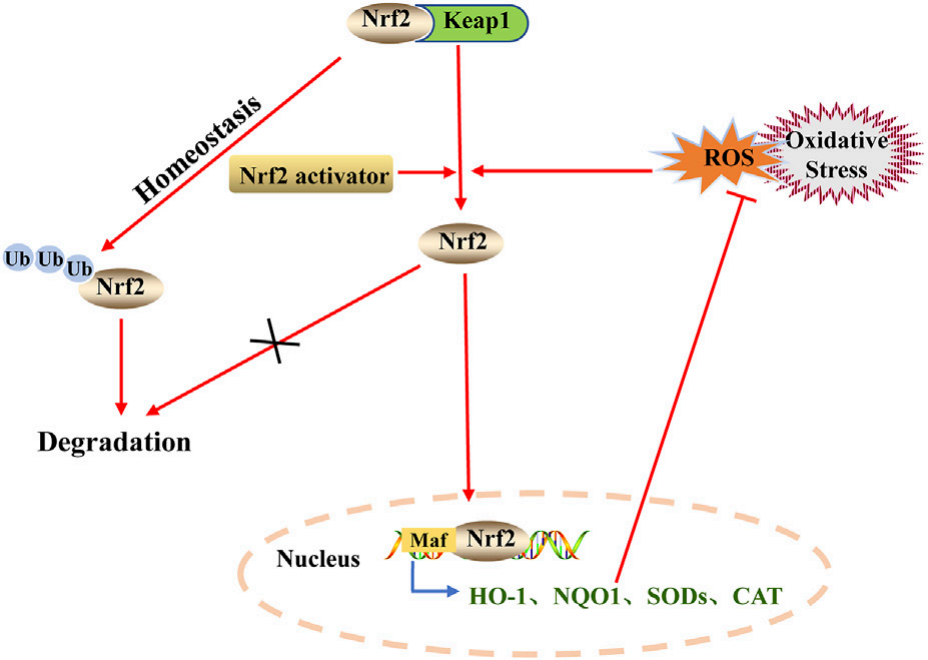
Regulation of Nrf2 degradation and activation. Under normal physiological conditions, Keap1 in the cytoplasm binds to Nrf2, which is ubiquitinated and degraded by the proteasome pathway. Under oxidative stress, the Nrf2-Keap1 complex is dissociated and Nrf2 is transferred to the nucleus. In the nucleus, Nrf2 binds to downstream antioxidant response elements (ARE) and activates the transcription of antioxidant genes.

Nrf2 is a multifaceted transcription factor that alleviates oxidative damage and inhibits inflammatory response (Ahmed et al., 2017). Under physiological conditions, Nrf2 binds to its inhibitor kelch-like ECH-associated protein 1 (Keap1) in the cytoplasm. Keap1 forms part of an E3 ubiquitin ligase, which mediates rapid ubiquitination and subsequent degradation of Nrf2 by the proteasome (Dinkova-Kostova et al., 2017). Keap1 is an adaptor protein for cullin 3 (CUL3)-based E3 ubiquitin ligases and forms a complex with CUL3 and RBX1 to regulate Nrf2 degradation (Kobayashi et al., 2004). The Keap1-CUL3-RBX1 E3 ubiquitin ligase complex functions to correctly orientate the Nrf2-bound Keap1 to facilitate ubiquitination of Nrf2. Once the ubiquitin chain reaches four ubiquitins in length, the polyubiquitinated protein becomes a new substrate for degradation by the 26S proteasome (Pierce et al., 2009; Baird and Yamamoto, 2020). Some reactive cysteine residues (Cys151, Cys273, and Cys288), as sensor proteins of Keap1, may modulate the Keap1-CUL3-RBX1 complex dissociation to promote Nrf2 stabilization in oxidative stress response (Zhang and Hannink, 2003). When stimulated by endogenous or exogenous oxidative stress, an intricate molecular mechanism facilitated by sensor cysteines within Keap1 allows Nrf2 to escape ubiquitination and enter the nucleus, where it binds to downstream antioxidant response elements (ARE) and activates the transcription of antioxidant genes, including heme oxygenase-1 (HO-1), NAD(P)H-quinone oxidoreductase 1 (NQO1), superoxide dismutase (SOD), and catalase (Figure 1) (Dinkova-Kostova et al., 2017; Baird and Yamamoto, 2020). In addition, the upregulation of Nrf2 signaling limits NF-κB activation, leading to inhibition of the excessive production of pro-inflammatory cytokines and chemokines, thereby mitigating signs of pulmonary inflammation (Mohamed et al., 2021). p300/CBP-associated factor (PCAF), a histone acetyltransferase, enhances the transcriptional activity of NF-κB (Sheppard et al., 1999). The inhibition of inordinately enhanced PCAF could mitigate fibrosis by redressing the aberrant balance between inflammatory signaling and antioxidant response through the modulation of NF-κB and Nrf2 (Chung et al., 2019). Recently, studies have demonstrated that a variety of natural medicines can reduce oxidative stress and inflammatory injury by activating the ALI/ARDS Nrf2 pathway (Liang et al., 2019; Liang et al., 2022). We focused on the relationship between inflammation and oxidative stress and the pathogenesis of ALI/ARDS. Moreover, we equally assessed the protective effect of the Nrf2 antioxidant pathway activated by several natural medicines used as therapy for ALI/ARDS.

## 2 Role of inflammation and oxidative stress in acute lung injury/acute respiratory distress syndrome

### 2.1 The vicious cycle of excessive inflammation and oxidative stress in acute lung injury/acute respiratory distress syndrome

**FIGURE 2:**
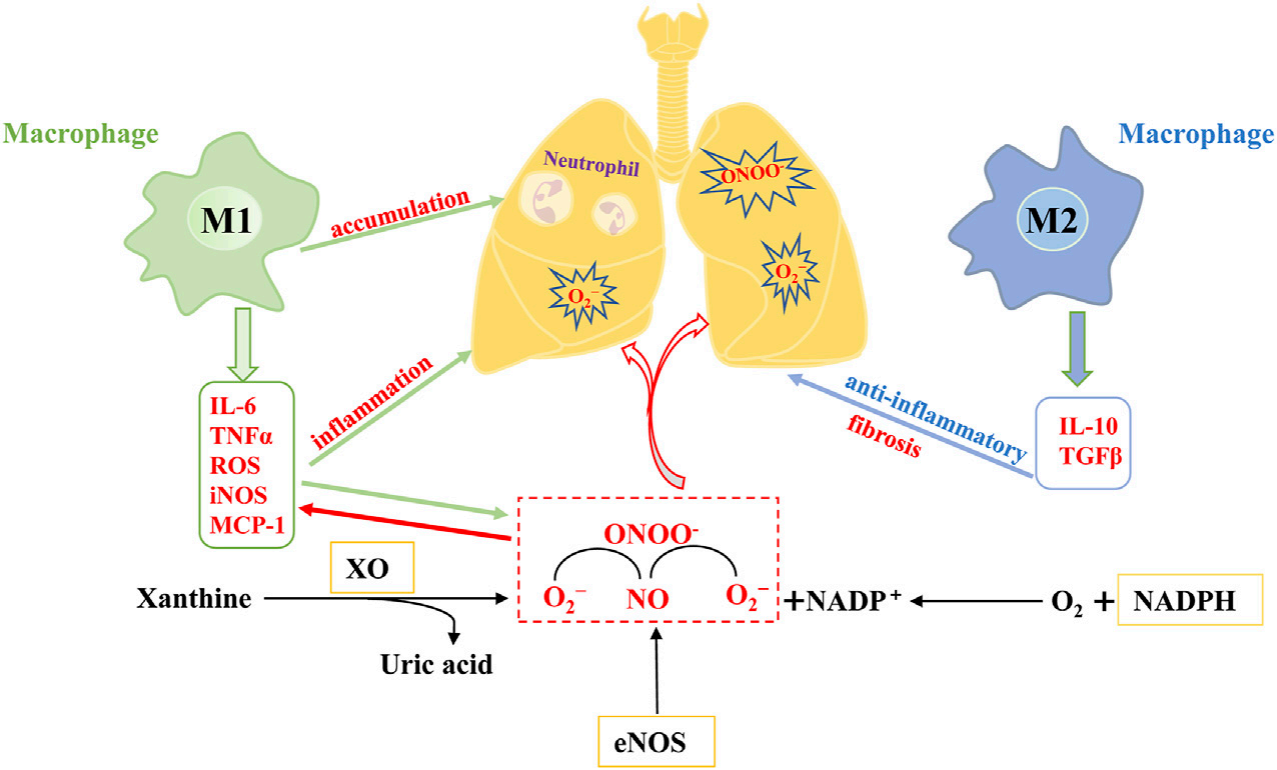
Mechanism of excessive inflammation and oxidative stress in ALI/ARDS. When lung endothelial cells are damaged, NADPH oxidase promotes the production of the superoxide anion (O^2−^), and xanthine oxidase (XO) catalyzes the oxidation of purine substrates to produce uric acid and O^2−^. In addition, NO derived from endothelial nitric oxide synthase (eNOS) forms highly reactive peroxynitrite with O^2−^ (ONOO-), exacerbating nitrification and oxidative damage in the lungs. Under the stimulation of various inflammatory and oxidative factors, macrophages polarize into M1 and M2 types. M1 macrophages secrete pro-inflammatory factors such as IL-6, MCP-1, and TNFα and aggravate neutrophil infiltration, leading to severe acute inflammatory injury of the lung. M2 macrophages secrete IL-10, TGFβ, and other factors conducive to inflammatory control, but the excessive production of these factors will aggravate the process of pulmonary fibrosis. In conclusion, the vicious cycle formed by excessive inflammation and oxidative stress intensifies the pathogenesis of ALI/ARDS.

The pathogenesis of ALI/ARDS is characterized by an excessive and uncontrolled inflammatory response of alveolar epithelial cells and capillary endothelial cells caused by a series of infectious or non-infectious factors (Tu et al., 2021). Pro-inflammatory cytokines, such as tumor necrosis factor-α (TNF-α), interleukin-1 *β* (IL-1β), and interleukin-6 (IL-6), cause injury and apoptosis of endothelial and epithelial cells in the pathogenesis of ALI, thereby damaging the alveolar-capillary barrier (Tu et al., 2021). Pulmonary vascular endothelial cells can inhibit inflammation and coagulation, recruit immune cells, and regulate leukocyte extravasation in inflammatory sites (Andonegui et al., 2002). Once the endothelial barrier is impaired, chemokines are secreted, and neutrophils and macrophages are recruited. This exacerbates a positive feedback loop of inflammation leading to an increased endothelial permeability through excessive production of oxygen free radicals (Goldenberg et al., 2011; Wu et al., 2021a). Macrophages are important effector cells involved in the exudative, hyperplastic, and fibrotic stages of ALI/ARDS. They are located at the site of tissue injury and then activated by mediators in the tissue microenvironment, polarizing them into M1 macrophages with pro-inflammatory/cytotoxic effects and M2 macrophages with anti-inflammatory/wound repair abilities (Huang et al., 2018; Shapouri-Moghaddam et al., 2018). In the acute exude stage of ALI/ARDS, pulmonary macrophages are M1-polarized and can release TNF-α, IL-6, nitric oxide (NO), and reactive oxygen species (ROS). These pro-inflammatory factors induce the recruitment of neutrophils, and the excessive accumulation of pro-inflammatory factors and neutrophils aggravates the inflammation of the lung (Patel et al., 2017; Huang et al., 2018). After the exudative stage, ALI/ARDS progresses to the hyperplasia repair stage and the phenotype of the host and recruits pulmonary macrophage changes from M1 to M2. M2 macrophages then activate anti-inflammatory signals, enhance the expression of anti-inflammatory factors IL-10 and TGF-β, and control pulmonary inflammation by clearing off apoptotic neutrophils at the inflammatory site, thereby promoting repair of the lung tissue (Figure 2) (Herold et al., 2011; Huang et al., 2018). M2 macrophages also inhibit the expression of inducible nitric oxide synthase (iNOS), a molecule associated with M1 macrophages, thereby preventing the production of ROS (Johnston et al., 2012). However, excessive M2 polarization leads to a pathological fiber proliferation response and pulmonary fibrosis during ALI/ARDS fiber proliferation (Xiang et al., 2016). In the pathogenesis of ALI/ARDS, the damaged lung endothelia/epithelia and pro-inflammatory cytokines and destructive oxidants produced by the recruited leukocytes directly cause tissue damage. Some oxidants (such as ROS) act as inflammatory signaling molecules to activate NF-κB, NLRP3, and other inflammatory pathways, consequently aggravating lung inflammation (Yang et al., 2019). Therefore, the prevention of lung endothelial/epithelial injury and the balance of M1/M2 macrophage phenotypic transformation mitigates the inflammatory and oxidative damage in ALI/ARDS pathology.

### 2.2 Regulation of multiple oxidases in acute lung injury/acute respiratory distress syndrome

#### 2.2.1 NADPH oxidase (NOX)

NOX is an oxidase-stimulating enzyme that catalyzes the reduction of oxygen to superoxide anion (O_2_^−^) through electron transfer. Therefore, it is often referred to as a “professional ROS producer” (Figure 2) (Ago et al., 1999). The NOX family consists of seven distinct isomeric compounds, of which NOX1, NOX2, and NOX4 are expressed in the vascular system and are associated with ROS-mediated vascular endothelial cell damage (Pendyala et al., 2009). In a hyperoxia-induced ALI mouse model, the loss of NOX1 reduced ROS production in the lung. Further studies showed that ROS produced by NOX1 activated the mitogen-activated protein kinase (MAPK) pathway in the lung tissue of mice during hyperoxia, thereby enhancing the phosphorylation of JNK and ERK. This leads to cell death and subsequent lung injury of lung endothelial and type II alveolar epithelial cells (Carnesecchi et al., 2009). Nuclear factor kappa B (NF-κB) mediates the expression of various inflammation-related genes. In ALI cell models, NF-κB activates the transcription of NOX1 in TNFα -stimulated A549 cells, thereby exacerbating lung injury. Targeting the NF-κB/NOX1/ROS signaling axis could be a potential strategy for further prevention and treatment of ALI (Wu et al., 2021b). In the lipopolysaccharide (LPS)-induced ALI mouse model, LPS promoted NOX2-mediated ROS production in the pulmonary vascular endothelial cells of mice by acting on Toll-like receptor4 (TLR4). NOX2-derived ROS activate STIM1, causing Ca^2+^ overload in pulmonary vascular endothelial cells, and exacerbating pulmonary vascular permeability and lung inflammation. ROS-driven Ca^2+^ signaling promotes vascular barrier dysfunction. The TLR4/NOX2/STIM1 mechanism provides a possible therapeutic target for limiting sepsis-induced ALI (Gandhirajan et al., 2013). In addition, administering NOX2-inhibitor in LPS-induced ALI mice improved the survival rate of ALI model mice, and drastically reduced the production of pulmonary ROS as well as pulmonary inflammatory factors (such as MCP-1, TNF-α, IL-17A) (Fisher et al., 2019; Nadeem et al., 2019). In a mouse model of ALI induced by cecal ligation puncture (CLP), administering a NOX2 inhibitor combined with antibiotics significantly reduced polymorphonuclear leukocyte (PMN) infiltration, pulmonary edema, and oxidative lung injury (Fisher et al., 2021). Clinical studies have shown that certain drugs (estrogen, NA-11) inhibit lung damage from NOX2-dependent oxidative bursts, and short-term estrogen administration mitigates pulmonary endothelial dysfunction, making them a promising option in the treatment of COVID-19 (Amara et al., 2021; Youn et al., 2021). In addition, researchers transfected NOX4 siRNA in CLP-induced ALI mice and found that NOX4 knockdown inhibited the activation of the CaMKII/ERK1/2/MLCK oxidation pathway, restored the expression of tight junction proteins ZO-1 and occludin in the pulmonary endothelial cells of mice, and maintained the integrity of the pulmonary endothelial cell barrier. In addition, these findings were verified in cultured primary human pulmonary microvascular endothelial cells (Jiang et al., 2020). *In-vitro* and *in-vivo* experiments of LPS-induced ALI demonstrated that treating ALI mice with antioxidants or NOX4 siRNA inhibited LPS-induced glycolysis and the production of inflammatory cytokines (Yuan et al., 2022). In a ventilator-induced lung injury (VILI) mouse model, administering NOX4 inhibitors or NOX4 knockout (NOX4 KO) reduced the degree of lung injury (Lee et al., 2022). Clinical studies have shown that an increased plasma NOX4 level was associated with weaning failure and 28-day mortality in patients with mechanical ventilation (Hong et al., 2020). In conclusion, NOX inhibitors show a protective effect against ALI/ARDS (caused by hyperoxia, sepsis, and ventilator-induced) by inhibiting the excessive production of ROS.

#### 2.2.2 Nitric oxide synthase (NOS)

Neuronal NOS (nNOS), endothelial NOS (eNOS), and iNOS are all expressed in the lungs and participate in oxidative and nitrifying stress responses (Fagan et al., 1999). In vascular tissues, eNO derived from eNOS induces vasodilation and plays an anti-inflammatory, anti-thrombotic, and anti-proliferative role (Yu and Liu, 2018). In the study of sepsis and hyperbaric oxygen-induced ALI mice, it was found that statins (such as simvastatin and pravastatin) mitigated pulmonary microvascular permeability and edema by upregulating eNOS (Bao et al., 2018; Ren et al., 2021). However, eNO also forms highly reactive nitrite peroxide (ONOO-) with O^2−^. This activates the nitroprotein RhoA through increased oxidative and nitrosative stress, and induces the uncoupling and redistribution of eNOS in mitochondria, thereby resulting in endothelial barrier dysfunction and lung injury (Figure 2) (Gross et al., 2015; Wang et al., 2021). In ALI mice induced by bleomycin (ITB), iNOS induced the recruitment of macrophages to the lungs during inflammation as well as macrophage polarization, thereby aggravating lung inflammation and oxidative damage (Golden et al., 2021). In addition, in a sepsis-induced ALI sheep model, researchers demonstrated that the lung function of sheep was significantly impaired, manifested by a gradual decrease in the oxygenation index and a consequent increase in pulmonary shunt fraction. These changes were related to an increased early expression of eNOS and iNOS, although the expression of early nNOS remained unchanged (Lange et al., 2010). Early inhibition of iNOS and late blocking of nNOS mitigates the damaging effects of inflammatory, oxidative, and nitrifying stress in ALI (Enkhbaatar et al., 2003; Zheng et al., 2019a). Therefore, the role of the three different subtypes of NOS in the development of ALI is controversial. The selective inhibition of NOS isomers and the study of their activity at different stages will provide new strategies for the treatment of ALI/ARDS.

#### 2.2.3 Xanthine oxidase (XO)

XO catalyzes the oxidation of purine substrates, such as xanthine and hypoxanthine, to produce uric acid and ROS. XO is one of the main oxidases that produce ROS during inflammatory conditions and oxidative stress (Figure 2) (Nomura et al., 2013). A clinical study involving the determination of plasma xanthine and hypoxanthine levels in patients with ARDS demonstrated that both substrates of XO were higher in all patients with ARDS, and plasma hypoxanthine levels were significantly higher in non-survivors of ARDS compared to levels in survivors. Therefore, the researchers hypothesized that non-survivors of ARDS experienced higher levels of oxidative stress damage than survivors (Quinlan et al., 1997). In rats with ALI induced by cytokines IL-1 and IFN-γ, lung inflammation and injury were involved in the activation of xanthine oxidoreductase (XOR) in newly recruited mononuclear phagocytes (MNP), and ROS produced by XOR in MNP exacerbated pulmonary inflammatory cell recruitment, oxidative stress, and alveolar cell apoptosis (Wright et al., 2004). Febuxostat, a potent XO inhibitor, protected rats from LPS-induced lung inflammation in a dose-dependent manner. This was manifested by a decreased TNF-α level, a decreased malondialdehyde (MDA) level (increased anti-oxidation), and increased SOD activity in the lung tissue of rats (Fahmi et al., 2016). These findings reveal the vital role of XO/XOR in ROS-mediated ALI/ARDS.

#### 2.2.4 Cytochrome P450 (CYP)

CYP is a group of isoenzymes with ferroprotoporphyrin as an auxiliary group. It catalyzes the oxidation of exogenous compounds in humans, regulates oxidative stress, and influences vascular permeability and inflammation. Different subtypes of CYP affect the pathogenesis of ALI (Stading et al., 2021). In CYP1A1^−/−^ deficient mice, CYP1A1 knockout enhanced LPS-induced ALI by inducing pulmonary edema, neutrophil infiltration, and destruction of lung parenchyma; meanwhile, TNF-α, IL-1β, IL-6, and NO levels were elevated, and these impairments were mediated by the overactivation of NF-κB and iNOS (Tian et al., 2020). In mice with acute hyperoxic lung injury, the expression of genes and proteins associated with DNA oxidative damage in the lungs of CYP1A −/− mice was increased, resulting in impaired DNA repair pathways (Lingappan et al., 2017). Compared with wild-type (WT) mice, CYP1A1^−/−^ mice experienced more episodes of severe pulmonary edema and neutrophil infiltration after hyperoxia induction (Lingappan et al., 2014). In addition, elevated lipid peroxidation, neutrophil infiltration, and IL-6 and TNF-α expression levels were observed in the lungs of CYP1A2^−/−^ mice under hyperoxia. The researchers concluded that CYP1A2 is vital in alleviating hyperoxia lung injury by reducing lipid peroxidation and oxidative stress (Wang et al., 2015). CYP1B1 and CYP1A belong to the same family; however, different from CYP1A1/1A2, CYP1B1 may cause hyperoxic toxicity. CYP1B1^−/−^ mice experienced a rapid decline in neutrophil levels when exposed to hyperoxia, thereby revealing that CYP1B1 is involved in early hyperoxia neutrophil recruitment of CYP1B1^−/−^ (Veith et al., 2018). In addition, isofuran is a lipid peroxidation product preferentially formed in hyperoxic conditions, thereby mediating hyperoxic toxicity. Compared with WT mice, the expression level of isofuran in lung tissues of CYP1B1^−/−^ mice after 24 h of hyperoxia was approximately 40% lower (Veith et al., 2018). In paraquat-induced ALI mice and cell models, inducing the expression of CYP450 and Nrf2 activated the detoxification pathway, reduced the accumulation of paraquat, mitigated pulmonary inflammatory cell infiltration, edema, and fibrosis, and increased the survival rate (Liu et al., 2019a). The isoenzyme of CYP450 is vital in the pathogenesis of ALI/ARDS. Thus, inducing CYP450 expression will affect the occurrence and development of ALI/ARDS and provide a target for the prevention and treatment of hyperoxic toxicity in ALI/ARDS.

## 3 Regulation of lung inflammation and oxidative stress with the Nrf2 pathway as the core

**FIGURE 3:**
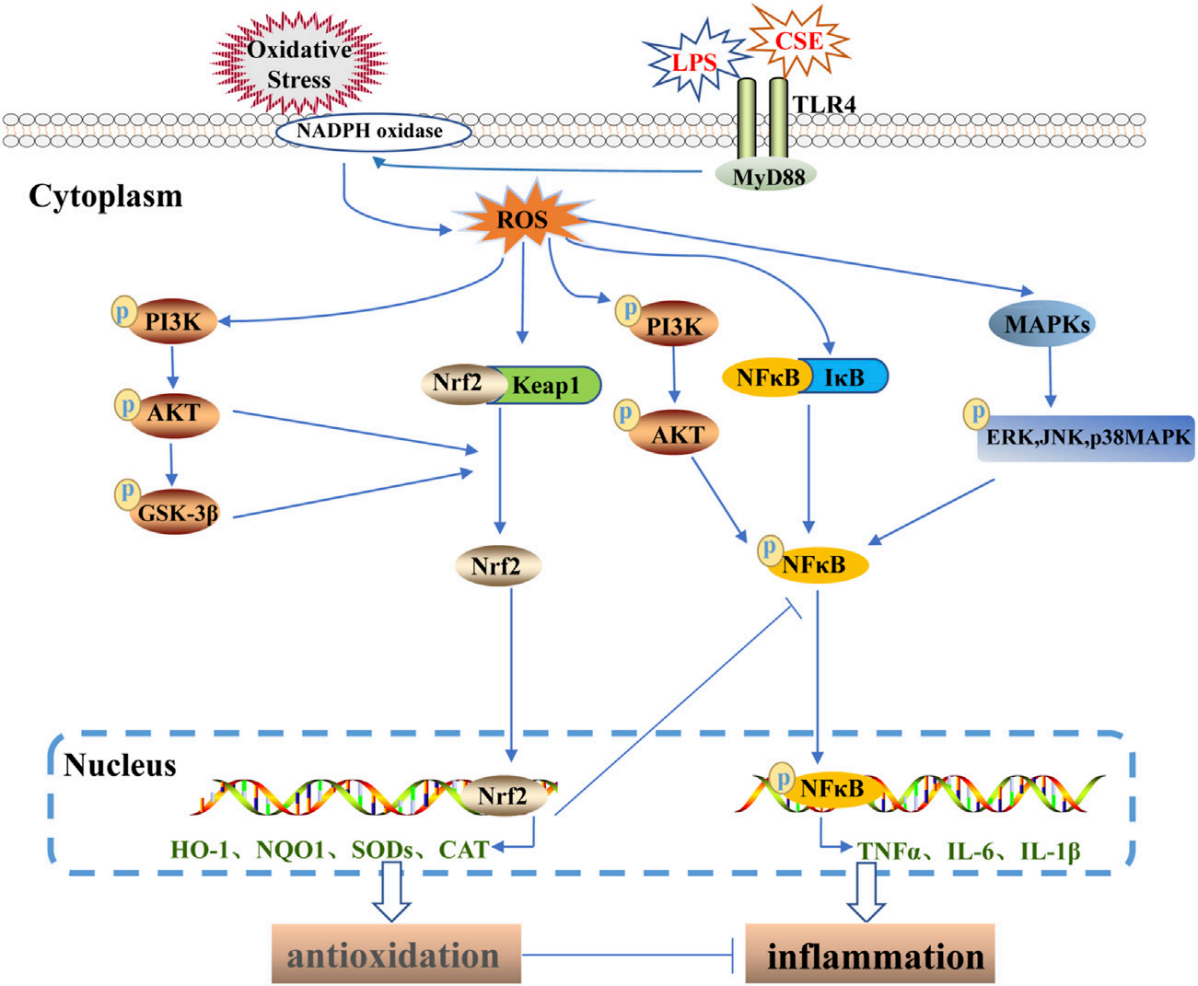
Regulation of lung inflammation and oxidative stress by Nrf2 signaling pathway crosstalk. Bacteria, viruses, harmful gases, and other stimuli induce excessive oxidative stress and inflammatory damage in the lungs. Animal studies have shown that Toll-like receptor-induced ROS production can be activated by endotracheal infusion of lipopolysaccharide (LPS) or inhalation of cigarette smoke (CSE) in mice. Excessive ROS directly activates the NFκB inflammatory pathway and the phosphorylation of AKT, ERK, JNK, and p38MAPK by activating the PI3K/AKT and MAPK inflammatory pathways, inducing the transfer of NFκB into the nucleus to promote the production of inflammatory factors (TNFα, IL-6, IL-1β). In addition, ROS directly promotes nuclear translocation of Nrf2 and induces nuclear transfer of Nrf2 through enhanced phosphorylation of AKT and GSK-3β, thereby promoting the expression of downstream antioxidant factors and inhibiting the NFκB inflammatory pathway. When ROS overproduction occurs, the Nrf2/ARE antioxidant pathway is unable to maintain a balance between oxidation and antioxidation, resulting in oxidative and inflammatory damage to the lungs.

Nrf2 has become a major target for the treatment of lung diseases (Liu et al., 2019b). With the occurrence of oxidative stress, the upstream signaling molecules of Nrf2 promote its entry into the nucleus to exert antioxidant and anti-inflammatory effects. Studies have shown that protein kinase B (AKT) regulates cell proliferation and apoptosis. Phosphorylation of AKT (P-Akt) is involved in nuclear Nrf2 localization and induces upregulation of nuclear Nrf2, HO-1, and NQO1, thereby reducing inflammatory oxidative stress and cell apoptosis in the lung (Fu et al., 2021). Excessive ROS leads to the accumulation of unfolded proteins in the endoplasmic reticulum. This directly triggers Nrf2/Keap1 dissociation and promotes Nfr2 nuclear input through PKR-like endoplasmic reticulum kinase (PERK) to maintain redox homeostasis (Cullinan et al., 2003). Under physiological conditions, glycogen synthase kinase 3β (GSK-3β) in the cytoplasm phosphorylates two degrons in Nrf2 to promote *β*-TRCP binding and ubiquitylation and its degradation (Mathur et al., 2018). Some studies have shown that LPS induces excessive ROS production in human alveolar epithelial cells, and some drugs inhibit Nrf2 degradation by inducing phosphorylation and inactivation of GSK-3β, resulting in nuclear transposition and activation, inhibition of LPS-induced excessive oxidative stress, and protection of alveolar epithelial cells (Ding et al., 2020). Adenosine monophosphate-activated protein kinase (AMPK) is also an enzyme that acts upstream of Nrf2. Phosphorylation of AMPK (p-AMPK) activates the Nrf2 antioxidant pathway, inhibits NLRP3 inflammasome, and reduces the oxidation and inflammatory damage of ALI (Huang et al., 2020). After Nrf2 enters the nucleus, it promotes the expression of its downstream antioxidant factors, thereby inhibiting excessive oxidative stress in the lung. However, it mitigates inflammatory lung damage by inhibiting inflammatory pathways. Nrf2 and NF-κB signaling cascades are closely related to oxidative and inflammatory diseases. Studies have shown that Nrf2 activation reduces the levels of inflammatory cytokines (IL-1β, IL-6, and TNF-α) by inhibiting the phosphorylation of IKK/IκB and nuclear translocation of the p65NF-κB subunit and mitigates inflammation and oxidative damage in ALI (Tang et al., 2021a). In intestinal ischemia-reperfusion (I/R)-induced lung injury, Nrf2 deficiency upregulates the expression of TLR4 and MyD88, and enhances I/R-induced lung inflammation and autophagy. In addition, Nrf2 deficiency induces cell death by down-regulating p-Akt (Yan et al., 2018). These findings revealed that the Nrf2/TLR4/Akt axis can be used as a therapeutic target for lung injury. In summary, crosslinking the Nrf2 antioxidant pathway and other signaling pathways reduces oxidative and inflammatory damage in the lungs (Figure 3).

## 4 The protective effect of natural medicines on various acute lung injury models by regulating the Nrf2 signaling pathway

### 4.1 The protective effect of natural medicines on lipopolysaccharide-induced acute lung injury by acting on Nrf2

Sepsis is a fatal condition with high incidence and is the main cause of ALI. LPS is a major component of the outer membrane of gram-negative bacteria. When in the lungs, LPS induces the activation of macrophages and recruitment of neutrophils, leading to inflammatory and oxidative damage in the lungs (Liu et al., 2017; Yang et al., 2019). Studies have shown that oridonin, xanthohumol, kirenol, PCP, and vincamine promote the phosphorylation of AKT, AMPK, and GSK3β and induce nuclear translocation of Nrf2, reduce ROS production in the lung tissue, inhibit the activation of the NLRP3 inflammasome, NF-κB and MAPK inflammatory pathways, and alleviate lung oxidation and inflammatory damage (Lv et al., 2017; Yang et al., 2019; Lin et al., 2021; Gan et al., 2022; Patangrao Renushe et al., 2022). Sinomenine, honokiol, isoalantolactone, and zerumbone, reduced the wet/dry (W/D) ratio of the lung by upregulating Nrf2/HO-1, decreased the levels of MPO and MDA, enhanced the activities of antioxidant enzymes such as SOD and GSH, and alleviated neutrophil infiltration in the lung (Leung et al., 2017; Yuan et al., 2018; Wang et al., 2020; Liu et al., 2021). By activating the Nrf2/HO-1 pathway, pterostilbene and corynoline mitigated the production of iNOS, COX-2, IL-1β, and TNF-α in the lung tissue, thus reducing interstitial edema and alveolar wall thickening (Liu et al., 2017; Zhang et al., 2020). Ethyl ferulate activates the AMPK/Nrf2 pathway, inhibits neutrophil infiltration and macrophage activation, and mitigates the production of pro-inflammatory mediators (iNOS, NO) and inflammatory cytokines (TNF-α, IL-1β, and IL-6) (Wu et al., 2021c). Cordycepin promotes Nrf2 nuclear translocation, inhibits the expression of NADPH and iNOS, reduces the level of superoxide in lung tissue, inhibits the expression of TLR4, and reduces the inflammatory response induced by TNF-α and IL-6 in the lungs (Qing et al., 2018). Recently, several studies have shown that ferroptosis is a type of regulatory necrosis that is more immunogenic than apoptosis and exacerbates the inflammatory response of ALI. Moreover, studies have demonstrated that panaxydol and obacunone inhibit lung injury caused ferroptosis through the activation of the Nrf2/HO-1 pathway, and inhibition of the production of inflammatory factors TNF-α, IL-1β, and IL-6 in the lungs (Li et al., 2021; Li et al., 2022). Therefore, the Nrf2 pathway activated by various types of natural medicines is involved in the prevention and treatment of septic ALI (Table 1).

**TABLE 1:** Natural medicines reduce ALI with Nrf2 as the core pathway.

Name	Major plants	Types	Dose and administration	Model	Ref
Oridonin	Rabdosia Rrubescens	diterpenoid	20/40 mg/kg (ip)	LPS-C57BL/6 mice	Yang et al. (2019)
Kirenol	Herba Siegebeckiae	diterpenoid	30/50/100 mg/kg (ip)	LPS-BALB/c mice	Lin et al. (2021)
Zerumbone	Zingiber zerumbet Smith	sesquiterpene	0/0.1/1 μmol/kg (ip)	LPS-ICR mice	Leung et al. (2017)
Isoalantolactone	Inula helenium	sesquiterpene	2.5/5/10 mg/kg (ip)	LPS-BALB/c mice	Yuan et al. (2018)
Panaxydol	Panax ginseng	polyacetylene	20 mg/kg (ip/iv)	LPS-C57BL/6 mice	Li et al. (2021)
Xanthohumol	hop plants	prenylflavonoid	10/50 mg/kg (ip)	LPS-C57BL/6 mice	Lv et al. (2017)
Myricetin	waxberry	flavonoid	100 mg/kg (IG)	CLP-C57BL/6 mice	Xu et al. (2021)
BNF	naturally occurring flavonoid	flavonoid	40 mg/kg (ip)	Hyperoxia-C57BL/6J mice	Callaway et al. (2020)
Cashew nut	Anacardium occidentale L	flavonoid, tocopherols	100 mg/kg (oral gavage)	AP-CD1 mice	Cordaro et al. (2020)
Formononetin	Radix Astragali	isoflavone	10/100 mg/kg (ip)	Hyperoxia-C57BL/6 mice	Chen et al. (2021)
Honokiol	Magnolia officinalis	compound	1.25/2.5/5 mg/kg (ip)	LPS-SD rats	Liu et al. (2021)
Ethyl ferulate	Chuanxiong	polyphenol	50 mg/kg (ip)	LPS-C57BL/6 mice	Wu et al. (2021c)
Resveratrol	Polygonum cuspidatum	polyphenol	30 mg/kg (ip)	CLP-Wistar rats	Wang et al. (2018)
Pterostilbene	Sandalwood grape	stilbene	10/20/40 mg/kg (ip)	LPS-BALB/c mice	Zhang et al. (2020)
Corynoline	Corydalis bungeana	isoquinoline alkaloid	15/30/60 mg/kg (ip)	LPS-BALB/c mice	Liu et al. (2017)
Sinomenine	Sinomenium acutum	isoquinoline alkaloid	100 mg/kg(ip)	LPS- ICR mice CLP- ICR mice	Wang et al. (2020; Song et al. (2021)
Vincamine	Vinca rosea	indole alkaloid	20/40 mg/kg (ip)	LPS-Swiss albino mice	Patangrao Renushe et al. (2022)
Cordycepin	Cordyceps sinensis	Purine alkaloid	1/10/30 mg/kg (iv)	LPS- Wistar rats	Qing et al. (2018)
PCP HPCP	Polygonatum cyrtonema Hua	polysaccharide	400/800 mg/kg (oral administration)	LPS-SPFKM mice	Gan et al. (2022)
LBP	Lycium barbarum	polysaccharide	100 mg/kg (IG)	Hyperoxia- C57BL/6 mice	Zheng et al. (2019b)
Emodin	Rhubarb	anthraquinone	25 mg/kg (Gavage)	AP-SD rats	Gao et al. (2020)

### 4.2 The protective effect of natural medicines on cecal ligation puncture-induced acute lung injury by acting on Nrf2

Studies have shown that intestinal microbiome dysregulation and intestinal barrier impairment through the “entero-lung axis” are associated with the progression of septic ALI (Tang et al., 2021b). In CLP-induced ALI mice, Myricetin activates the Nrf2/HO-1 antioxidant pathway, increases the activity of the antioxidant enzymes SOD and catalase, decreases the expression levels of iNOS and COX-2, and alleviates lung inflammation and oxidative damage (Xu et al., 2021). Resveratrol reduced pulmonary edema and inflammatory cell infiltration, activated the Akt/Nrf2 signaling pathway, inhibited IL-18, MDA, and caspase-3 expression in lung tissues of CLP group rats, and exerted anti-inflammatory, antioxidant, and anti-apoptotic effects (Wang et al., 2018). Sinomenine promotes Nrf2 transcriptional activity by activating aromatic hydrocarbon receptors, regulating intestinal flora homeostasis, and restoring the intestinal barrier, thereby inhibiting endotoxin transfer produced by the cecum and significantly reducing pulmonary edema and inflammatory factor levels (Song et al., 2021). Therefore, the Nrf2 pathway activated by natural medicines inhibits the progression of septic ALI by regulating the “enteric-lung axis” (Table 1).

### 4.3 The protective effect of natural medicines on hyperoxygen-induced acute lung injury by acting on Nrf2

Oxygen therapy and mechanical ventilation, widely used in patients with ARDS, increase the risk of hyperoxic exposure, leading to increased pulmonary microvascular permeability and inducing neutrophil infiltration and macrophage activation (Li et al., 2015). In addition, prolonged exposure to high oxygen intensified ROS production, leading to an imbalance in the oxidative response of the lungs (Li et al., 2015). Recent studies have shown that many natural drugs reduce the oxidative damage of lungs in animals with hyperoxic ALI. Lycium barbarum polysaccharide (LBP) activated the AMPK/Nrf2 signaling pathway, reduced MDA activity, reduced ROS production, and inhibited the expression of IL-1β and IL-6 (Zheng et al., 2019b). Moreover, formononetin activated the Nrf2/HO-1 antioxidant pathway, attenuated hyperoxy-induced pulmonary edema, enhanced the activity of antioxidant enzyme SOD, and induced polarization of M2 macrophages, thereby exerting an anti-inflammatory effect (Chen et al., 2021). Hyperoxia increased lung injury in Nrf2−/−mice, but Nrf2 transcription was significantly increased, and CYP1A1/1A2 activity was enhanced in Nrf2−/−mice pretreated with beta-naphthoflavone (BNF), thus alleviating lung damage caused by hyperoxia (Callaway et al., 2020). Although oxygen therapy and mechanical ventilation are the treatment methods for patients with ARDS, they can cause hyperoxic lung injury, and the addition of natural pharmaceutical ingredients prevents/treats hyperoxic lung injury by activating the Nrf2 pathway (Table 1).

### 4.4 The protective effect of natural medicines on acute lung injury induced by other factors by acting on Nrf2

ALI is a serious respiratory condition induced by endogenous and exogenous pathogenic factors, among which acute severe pancreatitis, intestinal ischemia-reperfusion injury, drowning, and other important factors are possible causes. In animal studies, lung injury during acute pancreatitis (AP) was characterized by edema and inflammatory cell infiltration. After treatment with Cashew nut, lung tissue injury was significantly alleviated. Researchers found that Cashew nut activates the Nrf2 pathway, inhibits NLRP3 pathway activation, and reduces the activities of MDA and MPO, thereby reducing inflammation and oxidative damage of lung tissue (Cordaro et al., 2020). In addition, in animal studies of AP, treatment with emodin increased nuclear translocation of Nrf2, inhibited NLRP3 inflammasome and NFκB inflammatory pathway activation, down-regulated TNF-α, IL-1β, and IL-6 expression, and effectively protected rats from AP-related lung injury (Gao et al., 2020). Both intestinal ischemia-reperfusion injury (IIR) and seawater drowning induce ferroptosis, resulting in the inhibition of intracellular antioxidant processes and the accumulation of ROS in mitochondria, leading to ALI. Ferroptosis can directly or indirectly inhibit glutathione peroxidase 4 (GPX4), which leads to intracellular antioxidant system damage and ROS accumulation in mitochondria, thereby causing cellular dysfunction (Probst et al., 2017). A study showed that ferroptosis can be inhibited by Nrf2 through regulating SLC7A11 and HO-1 (Dong et al., 2020). Many natural medicines (such as Astragaloside IV or Icariin) attenuated organ injury by inhibiting ferroptosis *via* the Nrf2/SLC7A11/GPX4 axis (Liu et al., 2022; Shao et al., 2022; Wang et al., 2022). Recent studies have shown that Nrf2 agonists inhibit the accumulation of lipid peroxides and ROS production and alleviate ALI caused by IIR and drowning by inhibiting ferroptosis (Qiu et al., 2020; Dong et al., 2021). Studies have demonstrated that natural pharmaceutical ingredients such as panaxydol and obacunone inhibit ferroptosis by activating the Nrf2/HO-1 pathway, thereby alleviating ALI. These findings reveal that natural medicine components inhibit ferroptosis by activating the Nrf2/HO-1 pathway, thus alleviating ALI (Li et al., 2021; Li et al., 2022). These findings also provide a direction for the study of natural medicines for treating ALI caused by IIR and seawater drowning (Table 1).

## 5 Summary and perspective

Nrf2 is the core transcription factor of cellular antioxidant response that protects the lungs from endogenous and exogenous injury. Under oxidative stress, the nuclear translocation of Nrf2 promotes the expression of several antioxidant enzymes downstream of Nrf2, such as HO-1, NQO1, SOD, and GCLC, thus reducing the oxidative damage in ALI. When ALI occurs, the activated Nrf2 signaling pathway inhibits the activation of the NF-κB inflammatory pathway and NLRP3 inflammasome and the expression of TNF-α, IL-6, IL-1β, TGF-β, and other inflammatory factors, therefore reducing the inflammatory response. In addition, Nrf2 regulates mitochondrial membrane potential, endoplasmic reticulum stress, ferroptosis, and the activity of multiple oxidases by crosslinking of many signaling pathways (Cullinan et al., 2003; Huang et al., 2020; Tang et al., 2021a; Li et al., 2021; Li et al., 2022). This stabilizes mitochondrial function and autophagy and inhibits severe inflammatory reactions and excessive production of ROS (vital in the prevention and treatment of ALI). Studies have demonstrated that natural drugs have low toxicity, few side effects, and remarkable clinical utility in the prevention and treatment of ALI through the activation of the Nrf2/ARE signaling pathway. In this study, the Nrf2 pathway was used as a target for the treatment of ALI, and we conclude that natural medicines such as flavonoids (Orientin and Myricetin), alkaloids (Sinomenine and Vincamine), terpenoids (Oridonin and Kirenol), polyphenols (Ethyl ferulate and Thearubigin), and polysaccharides (LBP and PCP) activate the Nrf2 pathway that is vital in the prevention and treatment of ALI induced by sepsis, IIR injury, AP, and hyperoxic conditions. Studies have illustrated that ROS at physiological concentrations is involved in the body’s immune defense; however, excessive ROS elimination is not conducive to the elimination of pathogens by immune cells (neutrophils, macrophages, and lymphocytes) (Ziltener et al., 2016). Therefore, mastering the dose and aging of natural drugs in the Nrf2 pathway and maintaining ROS homeostasis under oxidative stress is a subject for further in-depth research for the clinical prevention and treatment of patients with ALI/ARDS.
